# Thyrotoxicosis-Associated Hypercoagulability Leading to Concurrent Ischemic Stroke and Pulmonary Embolism in Graves’ Disease

**DOI:** 10.7759/cureus.99199

**Published:** 2025-12-14

**Authors:** Ammar K Alghanmi, Alaa Fahmi Khayat, Njood Ahmed Alhrkan, Ahmed Abdullah Alghamdi

**Affiliations:** 1 Neurology Department, King Saud Medical City, Riyadh, SAU

**Keywords:** graves’ disease, hypercoagulability, ischemic stroke, pulmonary embolism, thyrotoxicosis

## Abstract

Graves’ disease is the most common cause of endogenous hyperthyroidism, and it is increasingly recognized as a prothrombotic condition affecting both venous and arterial circulations.

We report a 27-year-old male with known but untreated Graves’ disease who presented with sudden-onset, left-sided weakness and facial asymmetry, with a National Institutes of Health Stroke Scale score of 9. Brain imaging confirmed multifocal right hemispheric ischemic infarctions with petechial hemorrhagic transformation, and post-contrast T1 black-blood vessel-wall MRI suggested inflammatory arteriopathy. CT pulmonary angiography identified acute pulmonary emboli involving the left upper and right lower lobe segmental pulmonary arteries. Laboratory testing demonstrated overt thyrotoxicosis with reduced Protein S levels, while Protein C, antithrombin III, autoimmune, and antiphospholipid panels were unremarkable. Therapeutic anticoagulation with enoxaparin 1 mg/kg twice daily was initiated and transitioned to apixaban 5 mg twice daily, along with carbimazole 20 mg/day and propranolol 40 mg three times daily. The patient improved clinically and was discharged with near-complete functional recovery. This case underscores that uncontrolled Graves’ disease can precipitate simultaneous arterial and venous thrombosis and highlights the importance of early recognition of thyrotoxicosis-associated hypercoagulability, prompt anticoagulation, and initiation of antithyroid therapy. Reassessment of thrombophilia after achieving euthyroidism is advised to guide anticoagulation duration.

## Introduction

Graves’ disease is the most common cause of endogenous hyperthyroidism and is increasingly recognized as a prothrombotic state that can involve both venous and arterial beds [1,2]. Excess thyroid hormone shifts hemostasis toward hypercoagulability through endothelial activation and increases in von Willebrand factor (vWF), factor VIII, and fibrinogen, coupled with reduced fibrinolytic activity; several studies show that these abnormalities improve after restoration of euthyroidism with antithyroid therapy [3-6].

Population-based studies and meta-analyses now demonstrate that patients with hyperthyroidism have a higher incidence of venous thromboembolism (VTE), including pulmonary embolism (PE). In a large Taiwanese cohort, hyperthyroidism conferred a >2-fold increased risk of PE over five years, and a nationwide Danish analysis showed an early excess of acute cardiovascular events after diagnosis [7,8]. Moreover, pooled data from systematic reviews report a significant association between hyperthyroidism and incident VTE [9,10].

Arterial thrombotic complications are also reported. Young adults with hyperthyroidism have an elevated risk of ischemic stroke on long-term follow-up, and thrombotic risk appears particularly pronounced early after diagnosis; treatment of hyperthyroidism mitigates this risk [2,8,11]. Mechanistically, experimental, observational, and Mendelian-randomization data support a causal link between higher thyroid hormone activity and procoagulant pathways [2,5,6]. Additionally, autoimmune thyroid disease may coexist with antiphospholipid antibodies (aPL), which, although often an epiphenomenon, can further tilt risk toward thrombosis in selected patients [12].

Against this backdrop, we report a young adult with newly diagnosed Graves’ disease who presented with simultaneous arterial ischemic stroke and acute pulmonary emboli. The case underscores the importance of recognizing thyrotoxicosis-associated hypercoagulability, instituting timely antithrombotic therapy alongside antithyroid treatment, and coordinating multidisciplinary care to reduce morbidity.

## Case presentation

A 27-year-old male with a known history of Graves’ disease, non-adherent to antithyroid therapy, presented to the emergency department with sudden-onset left-sided weakness and facial asymmetry. There was no preceding trauma, fever, headache, seizure, chest pain, or recent infection. He had no prior thromboembolic events, no family history of thrombophilia, and denied smoking, alcohol, or drug use.

On arrival, he was alert and oriented but anxious and tachycardic. Vital signs showed a blood pressure of 138/78 mmHg, heart rate of 122 beats/min, respiratory rate of 20 breaths/min, oxygen saturation of 97% on room air, and a temperature of 36.8 °C. Neurological examination revealed left facial palsy, left upper and lower limb weakness graded 3/5, decreased left-sided sensation, and left-sided inattention, yielding a National Institutes of Health Stroke Scale (NIHSS) score of 9. No signs of meningeal irritation were present. Cardiovascular and respiratory examinations were unremarkable except for tachycardia. No goiter, exophthalmos, tremor, or thyroid bruit was documented on admission, although the patient reported previous symptoms of thyrotoxicosis.

**Figure 1:**
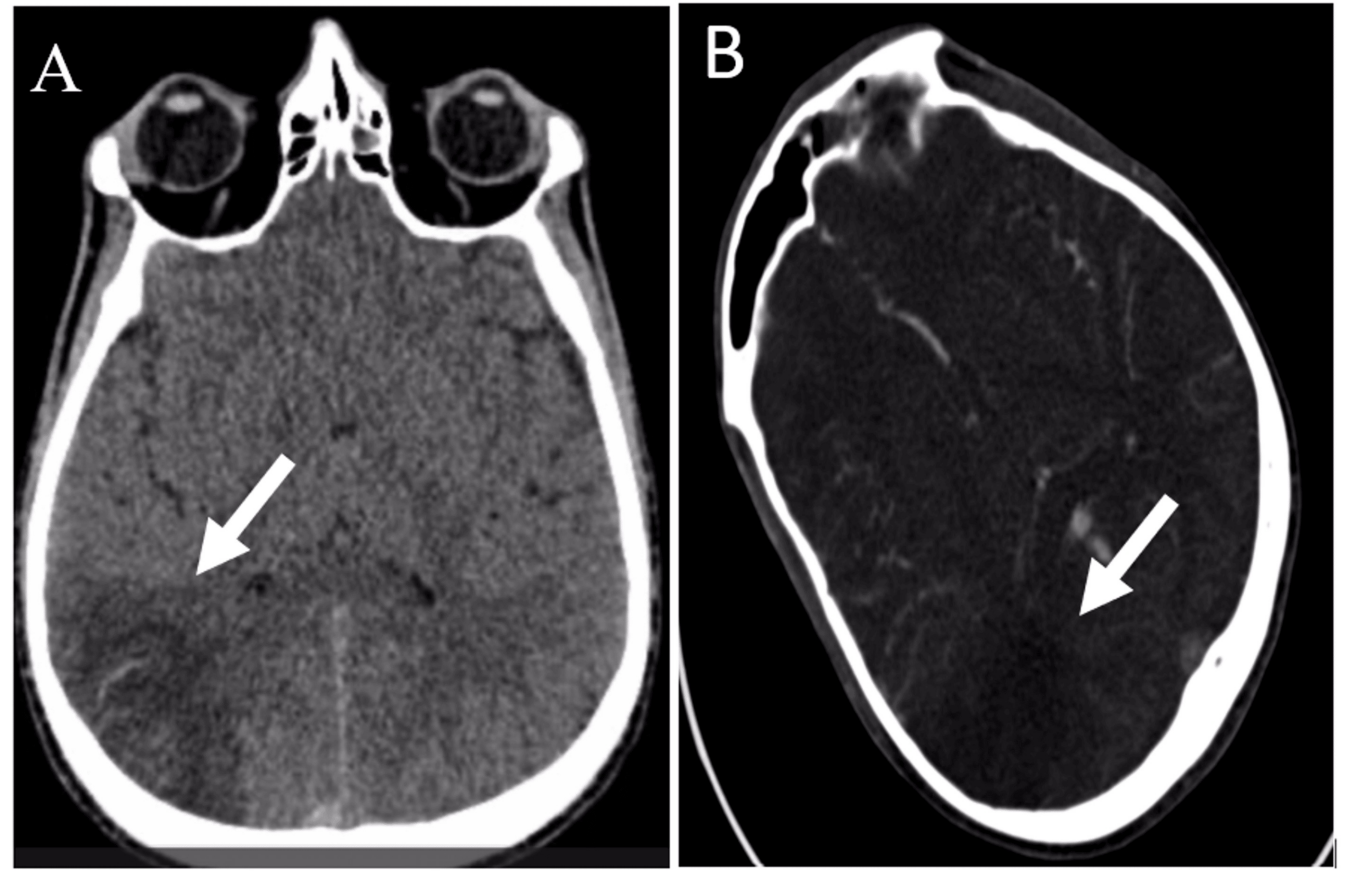
Non-contrast and contrast-enhanced CT brain showing acute right hemispheric infarction (A) Axial non-contrast CT image demonstrating a hypodense area in the right cerebral hemisphere (white arrow) consistent with an acute ischemic infarct, with loss of gray-white matter differentiation. (B) Axial contrast-enhanced CT/CT angiography showing an intraluminal filling defect in the right middle cerebral artery territory (white arrow), indicative of arterial thrombus with reduced downstream perfusion.

**Figure 2:**
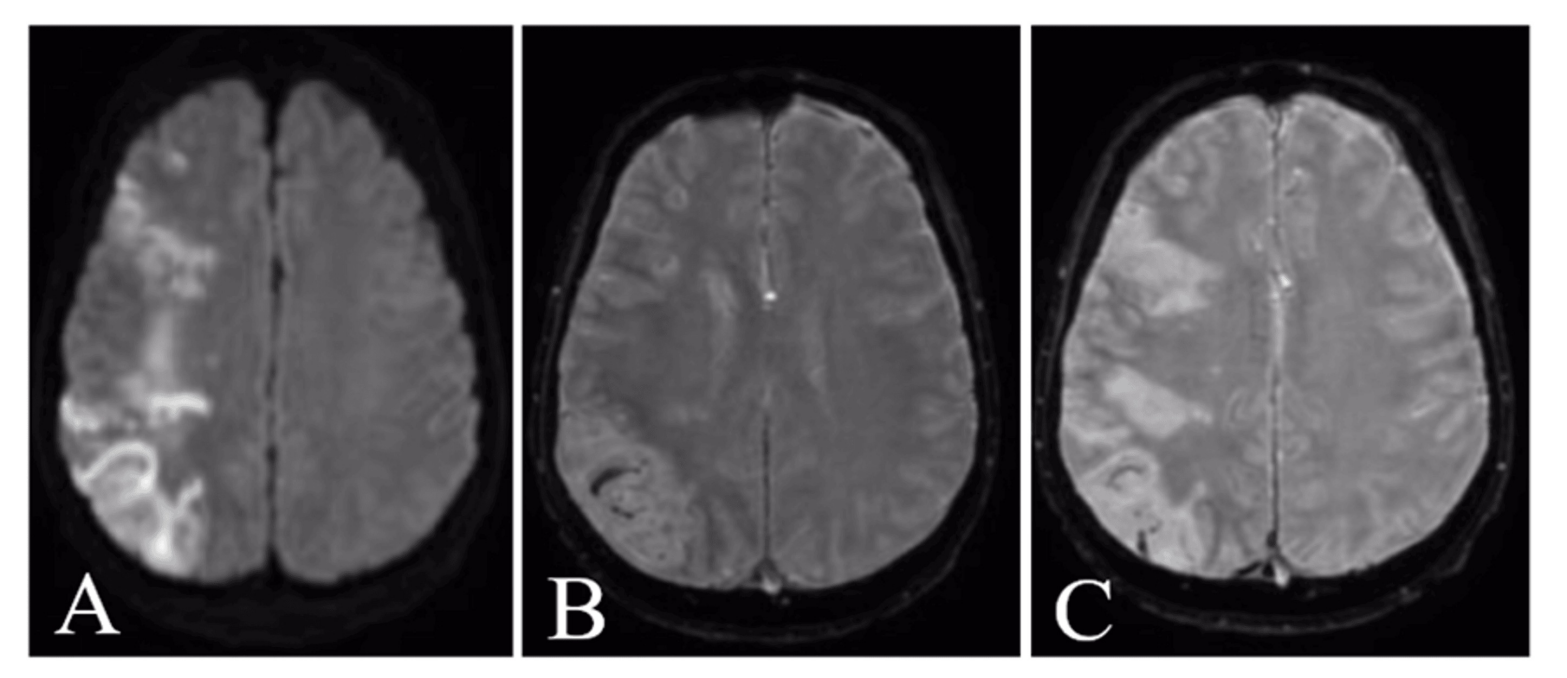
MRI brain demonstrating multifocal acute ischemic infarctions with hemorrhagic transformation (A) Axial diffusion-weighted imaging (DWI) showing areas of restricted diffusion in the right cerebral hemisphere, consistent with acute ischemic infarctions. (B) Corresponding apparent diffusion coefficient (ADC) map demonstrating low signal in the same regions, confirming true restricted diffusion. (C) Susceptibility-weighted imaging (SWI) revealing blooming artifacts within the infarcted areas, indicative of petechial hemorrhagic transformation.

Given the focal neurological deficits, an urgent non-contrast head computed tomography (CT) was performed, showing right parietal-occipital hypodensities (Figure 1). CT angiography revealed an intraluminal filling defect in the right middle cerebral artery circulation, suggestive of thrombus. Brain magnetic resonance imaging (MRI) confirmed multifocal subacute cortical and subcortical infarctions in the right cerebral hemisphere, with evidence of petechial hemorrhagic transformation on susceptibility-weighted imaging. Vessel-wall MRI demonstrated eccentric arterial wall enhancement predominantly on the right side, suggesting an inflammatory arteriopathy rather than classical atherosclerosis or embolic disease (Figure 2). Cervical and intracranial venous imaging excluded cerebral venous sinus thrombosis.

**Figure 3:**
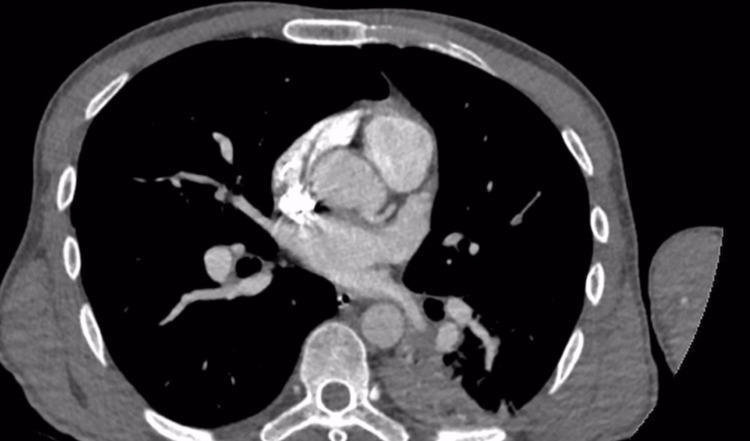
CT pulmonary angiography demonstrating a segmental infarct in the right lower lobe

In view of persistent tachycardia and mild dyspnea, a CT pulmonary angiography was performed, which demonstrated acute filling defects consistent with pulmonary emboli involving the left upper lobe segmental pulmonary artery and the right lower lobe segmental pulmonary artery (Figure 3).

Laboratory evaluation revealed biochemical evidence of overt thyrotoxicosis with suppressed thyroid-stimulating hormone (TSH) and elevated free triiodothyronine (FT3) and free thyroxine (FT4). Thyroid-stimulating hormone receptor antibodies (TRAb) were markedly elevated, confirming active Graves’ disease. A comprehensive thrombophilia and autoimmune screen was performed to explore the etiology of the concurrent arterial and venous thrombotic events. Protein S levels were reduced, whereas Protein C and antithrombin III levels were within normal limits. Antinuclear antibodies, extractable nuclear antigen panel, anti-double-stranded DNA antibodies, antiphospholipid antibodies, and lupus anticoagulant were negative, and infectious screening (HIV, hepatitis B and C, syphilis) was unremarkable. The full list of laboratory results is summarized in Table 1.

**Table 1:** Summary of key laboratory findings at admission TSH: Thyroid-stimulating hormone; FT3: Free triiodothyronine; FT4: Free thyroxine; TRAb: TSH receptor antibodies; ANA: Antinuclear antibodies; ENA: Extractable nuclear antigen; dsDNA: Double-stranded DNA antibodies; APS: Antiphospholipid syndrome; ACL: Anticardiolipin antibodies; β2GP1: Beta-2 glycoprotein I antibodies; ESR: Erythrocyte sedimentation rate; HIV: Human immunodeficiency virus; HBsAg: Hepatitis B surface antigen; HCV: Hepatitis C virus; ACE: Angiotensin-converting enzyme; Hb: Hemoglobin; ATIII: Antithrombin III

Test	Result	Reference Range
TSH	<0.01 mIU/L	0.53–4.94
FT3	10.22 pmol/L	2.43–6.01
FT4	26.06 pmol/L	9.01–19.05
TSH Receptor Antibodies (TRAb)	12.3 IU/L	<1.8
Hemoglobin A1c	9.0%	4.0–5.7%
ANA (IFA)	Negative	Negative <1.80
ENA Panel (SSA, SSB, RNP)	Negative	Negative
Anti-dsDNA	10.242 IU/mL	<80 negative
Anti-Cardiolipin IgM	1.485 MPL	<12.5
Anti-β2 Glycoprotein IgG	0.143 SGU/mL	0–20
Lupus Anticoagulant	Negative	Negative
Protein S	44%	55–140%
Protein C	96.7%	70–130%
Antithrombin III	104%	80–120%
Factor V Leiden (APC Resistance Test)	107.2 sec	120–300 sec
Factor XII	85%	60–150%
Homocysteine	0.80 μmol/L	4–15
Hb Electrophoresis	HbA 96.7%; HbA2 3.3%	HbA >95%; HbA2 <3.5%
ACE Level	52 U/L	9–67
HIV 1/2, HCV Ab, HBsAg, Syphilis	Negative	-
ESR	2 mm/hr	0–30 mm/hr

The patient was managed with antithrombotic therapy, antithyroid treatment, and supportive care. Given the coexistence of an ischemic stroke with petechial hemorrhagic transformation and acute pulmonary embolism, therapeutic anticoagulation with enoxaparin at 1 mg/kg twice daily was initiated and continued for five days before transitioning to apixaban 5 mg twice daily. Carbimazole was started at a dose of 20 mg/day to restore euthyroidism, and propranolol at 40 mg three times daily was prescribed to control adrenergic symptoms related to thyrotoxicosis. Multidisciplinary management involving the neurology, endocrinology, and hematology teams was provided. The patient showed progressive neurological improvement, with resolution of tachycardia and normalization of thyroid function parameters during follow-up.

At discharge, he exhibited significant clinical recovery, with mild residual left-sided weakness and near-complete return of functional independence. He was discharged on anticoagulation and antithyroid therapy with outpatient follow-up arranged for reassessment of Protein S levels after restoration of euthyroidism.

## Discussion

This case highlights a rare convergence of arterial ischemic stroke and pulmonary embolism (PE) in a young adult with active Graves’ disease and low Protein S, after exclusion of cardioembolic sources and systemic autoimmune conditions. The clinical picture fits a thyrotoxicosis-associated hypercoagulable state, in which excess thyroid hormone promotes endothelial activation, increases vWF/FVIII/fibrinogen, and suppresses fibrinolysis; importantly, these abnormalities tend to improve with restoration of euthyroidism [1-6]. Our patient’s laboratory profile, suppressed TSH, elevated FT3/FT4, strongly positive TRAb, and reduced Protein S, is consistent with that pathophysiology, whereas Protein C/antithrombin III were normal, and antiphospholipid/autoimmune screening was non-diagnostic, lowering the likelihood of alternative thrombophilic mechanisms [1,4-6,12].

Epidemiologic data show that hyperthyroidism raises the risk of venous thromboembolism, including PE, and is also associated with arterial ischemic stroke, particularly early after diagnosis [6-11]. Our patient’s simultaneous events emphasize that thyrotoxicosis can produce a bi-circulatory thrombotic phenotype, not merely venous disease. Recent reports further document PE occurring with Graves’ disease, reinforcing a causal connection between uncontrolled thyrotoxicosis and clinically significant VTE [7-10].

Beyond confirming multifocal infarctions and minor hemorrhagic transformation, vessel-wall MRI (VWI) suggested an inflammatory arteriopathy rather than classic atherosclerosis. Contemporary VWI literature underscores its diagnostic value for non-occlusive intracranial vasculopathies: concentric wall enhancement favors vasculitis, whereas eccentric patterns are more typical of atherosclerosis; enhancement dynamics can also guide follow-up and management [13,14]. In our context, young age, absence of conventional atherosclerotic risk, and an active systemic inflammatory-endocrine disorder, the VWI pattern supports secondary inflammatory endothelialopathy related to thyrotoxicosis rather than primary vasculitis, consistent with negative autoimmune serologies [12-14].

The low Protein S in our patient plausibly reflects an acquired deficiency linked to thyrotoxicosis, as described in mechanistic and clinical studies showing reductions in natural anticoagulants during hyperthyroidism with improvement after treatment [1,5]. Because acute illness, anticoagulation, and thyroid status can transiently alter Protein S levels, repeat testing after achieving euthyroidism is recommended to distinguish acquired from hereditary deficiency and to inform long-term anticoagulation strategy [1,6].

The coexistence of acute PE and ischemic stroke with petechial hemorrhagic transformation required careful balancing of thrombotic and hemorrhagic risks. In line with contemporary VTE guidance, therapeutic anticoagulation is indicated for acute PE, with direct oral anticoagulants (DOACs) preferred over vitamin K antagonists in the absence of contraindications [15,16]. Initiating therapeutic low-molecular-weight heparin (LMWH) followed by apixaban is clinically coherent in this setting and allows titratable anticoagulation early on while monitoring the small hemorrhagic component. Concomitantly, antithyroid therapy and beta-blockade address the causal endocrine driver and mitigate adrenergic symptoms; reduction of thyroid hormones is expected to rebalance hemostasis over time [1-6].

Cardioembolism was unlikely given the negative rhythm monitoring and echocardiography. Cerebral venous thrombosis (CVT) was excluded on neuroimaging, although thyrotoxicosis-related CVT is recognized in the literature and shares similar prothrombotic mechanisms [7-10]. Autoimmune vasculitides and antiphospholipid syndrome were not supported by serology or imaging features [12-14].

As in many acute presentations, certain laboratory values can be confounded by acute-phase effects and anticoagulation; confirming persistence or normalization after euthyroidism is essential for etiologic classification and for decisions on the duration of anticoagulation [1,5,6,16]. Serial VWI could further clarify whether arterial wall enhancement abates with endocrine control, strengthening the link between thyrotoxicosis and inflammatory arteriopathy [13,14].

This case underscores three practical messages: uncontrolled Graves’ disease can precipitate clinically significant thrombosis in both arterial and venous beds; VWI may help distinguish inflammatory arteriopathy from atherosclerosis in young patients with stroke and systemic endocrine activation; and a coordinated approach, timely full-dose anticoagulation for PE, antithyroid therapy plus beta-blockade, and structured follow-up to reassess thrombophilia, can achieve good neurological and systemic outcomes [1-6,13-16].

## Conclusions

Uncontrolled Graves’ disease can trigger a systemic hypercoagulable state with concurrent arterial and venous events, strengthened here by low Protein S and vessel-wall MRI features suggestive of inflammatory endothelialopathy. Prompt, coordinated care, therapeutic anticoagulation despite minor hemorrhagic transformation, plus antithyroid therapy and beta-blockade, can lead to meaningful recovery. Clinicians should actively screen for VTE and arterial complications in severe thyrotoxicosis, and repeat thrombophilia testing after euthyroidism to distinguish acquired from hereditary defects and to guide anticoagulation duration.
